# A Pilocytic Astrocytoma Mimicking a Clinoidal Meningioma

**DOI:** 10.1155/2014/524574

**Published:** 2014-03-13

**Authors:** Christopher S. Hong, Norman L. Lehman, Eric Sauvageau

**Affiliations:** ^1^Department of Neurological Surgery, The Ohio State University Wexner Medical Center, 410 W. 10th Avenue, N1022 Doan Hall, Columbus, OH 43210, USA; ^2^Department of Pathology, The Ohio State University Wexner Medical Center, 333 W. 10th Avenue, 4169 Graves Hall, Columbus, OH 43210, USA

## Abstract

Pilocytic astrocytomas and meningiomas are benign, primary brain tumors that may involve the optic tract. Classically, the presence of a dural “tail” sign may differentiate a meningioma from other intracranial lesions. In this report, we describe a mass with the typical appearance of a clinoidal meningioma on magnetic resonance imaging (MRI) but postoperatively diagnosed as a pilocytic astrocytoma. This case illustrates the rare occurrence of a pilocytic astrocytoma mimicking a meningioma on MRI.

## 1. Background

Pilocytic astrocytomas are benign, World Health Organization (WHO) grade I gliomas that most frequently arise in adolescents and young adults and usually involve the cerebellum, hypothalamus, and optic pathways. Radiographically, they are relatively well demarcated and strongly enhance on MRI as they are well vascularized and often exhibit glomeruloid vascular proliferation. Although rare, pilocytic astrocytomas can originate from the optic pathway, anterior or posterior to the optic chiasm. These lesions have a strong association with neurofibromatosis type 1 (NF1) and are estimated to occur in 15% of all patients with this inherited syndrome [1].

In contrast, meningiomas are benign growths of arachnoid cap cells, usually found at sites of dural reflections within the skull, and classically exhibit a homogenously enhancing thickening of the dural margin with peripheral tapering on MRI, known as the “tail” sign [2]. Because of these characteristic features, meningiomas are usually readily identified based on clinical and radiographic findings alone, often precluding a need for tissue biopsy prior to treatment.

In this case report, we describe a young patient whose clinical and radiographic presentation strongly supported a diagnosis of a meningioma but upon neurosurgical intervention was found to have a pilocytic astrocytoma. We describe the clinical course of our patient and highlight the unusual features that were subsequently found.

## 2. Case Report

**Figure 1:**
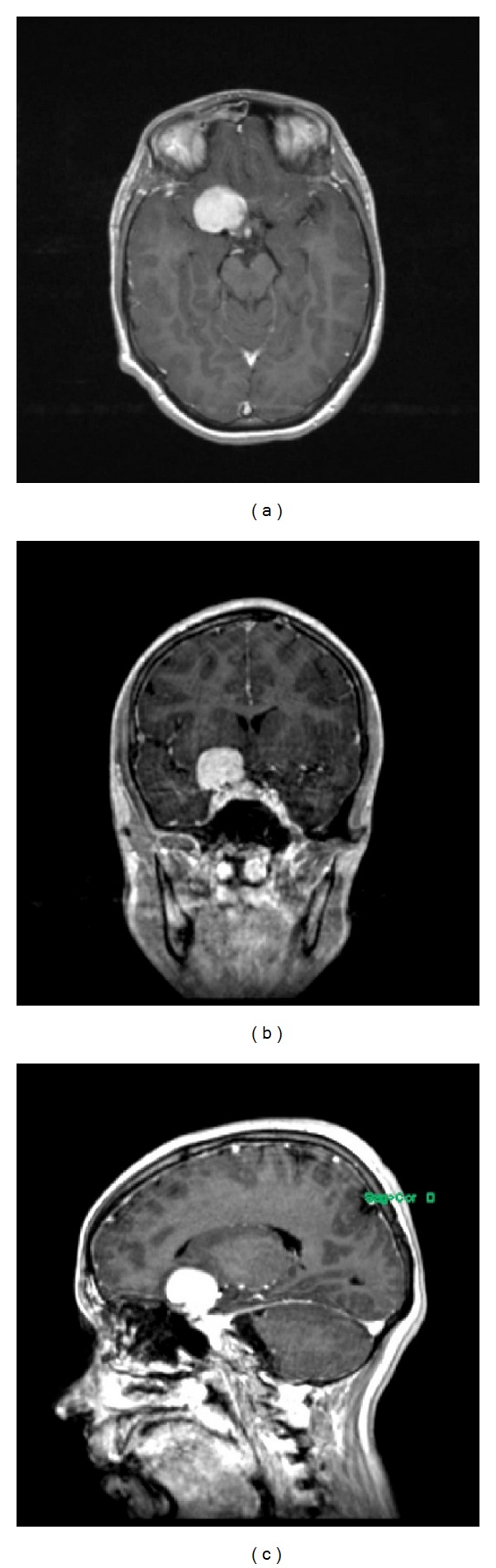
(a) Axial, (b) coronal, and (c) sagittal T1-weighted MRI showing a homogenously enhancing lesion, measuring 2.9 × 3.0 × 2.0 cm in the right clinoidal region. There is probable extension into the right optic canal, sphenoid sinus, right temporal fossa, and possibly right cavernous sinus, suggestive of a right clinoidal meningioma.

An 18-year-old female, 36 weeks into pregnancy, presented to ophthalmology with poor right-sided vision evolving over few years. Her exam was notable for right optic nerve pallor and atrophy. A brain MRI without contrast due to her pregnancy showed a right supraclinoid mass measuring 2.5 × 2.2 × 2.5 cm, for which she was referred for neurosurgical evaluation. No other neurological deficits were present on examination. Considering the stability of the visual deficit and the late gestational age, a contrast study was postponed until after her delivery. The subsequent MRI confirmed an extra-axial mass in the right clinoid region with homogenous enhancement and interval growth of the lesion to 2.9 × 3.0 × 2.0 cm (Figure 1). There were mild mass effect upon the right aspect of the optic chiasm, evidence of a dural tail, encasement of the right internal carotid artery, and possible extension into the right cavernous sinus. Collectively, these findings radiologically suggested a diagnosis of a right clinoidal meningioma.

**Figure 2:**
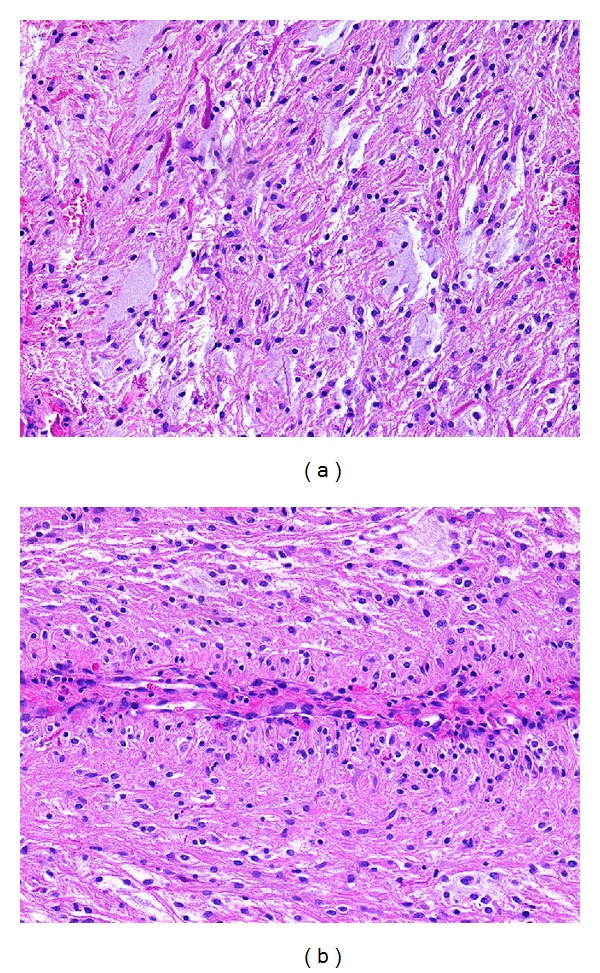
(a) The tumor consists of piloid cells and shows abundant mucin and Rosenthal fibers (upper left) (H&E stain; original magnification 200x). (b) A few perivascular pseudorosettes were noted, here in longitudinal section (H&E stain; original magnification 200x).

The patient subsequently underwent surgical resection via a right frontotemporal craniotomy. Intraoperatively, the frontal excursion of the tumor had a well-defined arachnoid plane, but further opening of the dura revealed tumor infiltration of the optic nerve, which was unexpected for a meningioma. A biopsy was taken from the superior truncal aspect of the tumor and sent to pathology for intraoperative diagnosis, which returned a diagnosis of pilocytic astrocytoma. The final pathology report described a proliferation of piloid glial cells of low to moderate cellularity associated with abundant Rosenthal fibers, rare eosinophilic granular bodies, and microvascular proliferation mostly located at the periphery of the tumor (Figure 2). A few pilomyxoid features were noted, including abundant extracellular mucin, some perivascular pseudorosetting, and paucity of eosinophilic granular bodies. Collectively, the histologic findings confirmed a diagnosis of pilocytic astrocytoma, WHO grade I.

Postoperatively, the patient recovered without significant complications and reported improved overall vision at her one-month follow-up visit. The 6-month postoperative MRI showed no interval growth of the residual tumor, and the patient is currently receiving regular surveillance imaging.

## 3. Discussion

Numerous factors led us to initially believe that our patient's lesion was dural based. Radiographically, the MRI showed a uniformly enhancing lesion that seemed to communicate with the dural surface overlying the right clinoidal area. While there seemed to be extension into the optic canal, preoperative imaging did not suggest optic nerve infiltration. Clinically, our patient had no features of NF1 to support a tumor originating from the optic nerve. Furthermore, the accelerated interval growth of her lesion during pregnancy was consistent with known effects of gestational hormones on meningioma proliferation [3]. Intraoperatively, however, there were clear involvement and possibly origination of the tumor from the optic nerve, given the preserved arachnoid plane along its borders, which supported the final pathologic diagnosis of an astrocytoma.

A thorough review of the literature revealed only one other report of an optic pathway glioma mimicking a meningioma on preoperative imaging. Edwards et al. recently discussed a case of an 11-year-old child who presented with complaints of right-sided vision loss. On preoperative imaging, there were an enlarged right optic nerve and a homogenously enhancing, dural-based lesion that extended into the right temporal lobe [4]. During surgery, however, it was clear that the tumor involved white matter tracts, most likely derived from the right temporal lobe. Notably, while hyperintensity on T2-weighted MRI suggested infiltration into the optic tracts, this could not be visualized, intraoperatively. The final histologic diagnosis was a pilomyxoid astrocytoma.

Pilomyxoid astrocytomas were first described in 1999 and subsequently were recognized by the WHO in 2007 as a more aggressive, histological variant of pilocytic astrocytomas. On MRI, these tumors usually enhance homogenously within the chiasmatic-hypothalamic region. Unlike pilocytic astrocytomas, they exhibit T2 signal extension into surrounding deep white and grey matter, in contrast to our patient's radiographic presentation. Interestingly however, the pathology of our biopsy sample did report limited pilomyxoid features. Generally, less than 5% of pilocytic astrocytomas progress towards malignancy [5]. Still, our patient will continue to be closely monitored for signs of disease progression.

## 4. Conclusion

In this case report, we describe the clinical course of a patient who presented with clinical and radiographic signs, all suggestive of clinoidal meningioma. However, the gross appearance and histology of the lesion demonstrated a pilocytic astrocytoma. Therefore, physicians should consider a pilocytic astrocytoma in their differential diagnosis of any dural-based, enhancing lesion within the periclinoidal region.
